# Hospital Meetings, &c.

**Published:** 1898-02-26

**Authors:** 


### HOSPITAL MEETINGS, &C.

### WORKING MEN AND HOSPITALS.

The first of a series of meetings arranged by a joint committee of the Federation of Working Men's Social Clubs and the Club and Institute Union was held at the offices of the London Chamber of Commerce on Wednesday, February 16th. There was a very large attendance of delegates from workmen's clubs, and Mr. HAROLD BOULTON, who was elected to the chair, stated that the joint committee had arranged four meetings on the following points: (1) To consider the better organisation of the sources of support to hospitals on the part of the working classes, and in particular of working men's clubs and similar bodies, express reference to be made under this head to the Hospital Saturday Fund. (2) Reforms in relation to the out-patient department. (3) The position of provident dispensaries and similar organisations in connection with the hospitals. (4) To discuss the question of the better management and organisation of the hospital system generally through the establishment of a Central Board or otherwise.

The CHAIRMAN first called upon Mr. R. B. D. Acland, chairman of the Hospital Saturday Fund, to make a statement as to the methods of his organisation.

Mr. ACLAND explained at the outset that what he meant by "hospital" was medical relief in whatever form it might take. He would include convalescent homes, nursing institutions, bodies for the supply of surgical appliances, and even ambulance work. After alluding with satisfaction to the abolition of the system of street collecting by the Saturday Fund, Mr. Acland declared that their experience had been that collecting sheets in workshops were far more effective than collecting boxes. Sometimes they had difficulties owing to the workmen, and sometimes through the obstructiveness of the employer, and bodies like those represented there to-night might be able to exercise their influence in order to have these difficulties removed. He was happy to say that since the abolition of the street collections the shop collections had shown steady increase. Coming to the question of distribution, Mr. Acland said that the Saturday Fund endeavoured to distribute their money in such a way that those who subscribed should get admission to the hospitals to which they subscribed. They also contributed to convalescent homes, and expended a portion of their funds on surgical appliances and on nursing institutions. They also reserved a sum of money for hospital letters over and above those they got in the ordinary course. The working classes said, "If we subscribe to hospitals we ought to have the same number of letters as if we were ordinary subscribers." That might be right or it might be wrong, but so long as the letter system existed the contention appeared to him to be a right one. As for himself, he believed the letter system to be bad. (Cheers.) He thought the proper passport to a hospital should be sickness and the need of help, and he hoped Mr. Sydney Holland would devote his great energy to the abolition of hospital letters.

Mr. HOLLAND here interposed and said that letters had not been abolished at the London, because they had not the building ready. He hoped, however, that in two years they would be a thing of the past as far as that hospital was concerned; meanwhile they had been abolished at Guy's, St. Bartholomew's, St. Thomas's, and Poplar.

Mr. ACLAND, continuing, described the Saturday Fund's system of assisting patients to obtain surgical appliances. The Fund purchased the appliance necessary in each case and handed it over to the person requiring it, who then became responsible for repayment to the Fund by instalments spread over a period of time. They also had recently formed an alliance with the St. John Ambulance Society, through whose agency they were giving instructions in "first aid" in workshops. They had also provided ambulances where no facilities existed for carrying injured people to the hospital, and further had made arrangements by which, on payment of part of the cost, they could undertake to remove any sick person within the metropolitan area to any hospital.

Mr. SYDNEY HOLLAND asked whether the abolition of letters at the London would affect the Saturday Fund grant, and to this Mr. ACLAND replied in the negative. Mr. Holland then stated that the Poplar and London Hospitals received more direct support from working men than they did through the indirect means of the Saturday Fund. He wished to know whether it would not be possible to divide London into districts and to arrange that the funds collected in each district should be apportioned to the local hospitals. He also submitted that giving classes in "first aid," providing street ambulances, and removing sick people to the hospitals, was not suitable work for the Saturday Fund to perform.

Mr. ACLAND replied that those who subscribed the money had a perfect right to use it in the manner they thought best, especially as the objects of the collection were clearly laid down beforehand. With regard to collecting for the local hospitals, the idea was, in the opinion of himself and his friends, not a practical one. As Sir Henry Burdett had pointed out, the greater number of the hospitals were within a mile of each other; and, besides, a large central organisation would give the working classes more power than a number of small individual subscriptions.

At this point a DELEGATE made a statement to the effect that collections on behalf of the Saturday Fund had been made for two or three years at the Woolwich Arsenal, but that none of the money had been sent in.

Mr. ACLAND: I know this case, and, to put it in brutal frankness, the money has been stolen. I do not see why this should be put down to the Saturday Fund.

Mr. HOLLAND: Has there been a prosecution?

Mr. ACLAND: No; they can't prosecute.

Mr. BUNN (secretary to the Hospital Saturday Fund): I know that our fund is not the only sufferer. (To the delegate): Don't you think this matter concerns yourselves; we are not there, you know.

The DELEGATE: It is a curious thing that no inquiry was made for three years.

Mr. BUNN: You know that we do not appoint the collector; it is you who do that.

In the course of further discussion, Mr. BUNN warmly defended the policy of the fund in connection with surgical appliances. They had, he said, done ambulance work in a way the St. John Society and Hospitals' Association had never attempted. By attending meetings in Deptford and Bermondsey, they found the sufferings of people who were carried in a cart to Guy's were intense, and so they provided the remedy. As for the idea of dividing London, it was impossible, and Mr. Holland knew it.

Mr. HOLLAND: I don't, really.

Mr. HOLLAND suggested that the sale of the hospital stamps, if properly taken up, would relieve the hospitals of the necessity of begging. Had the clubs ever considered the idea? Every hospital was in serious want, and the result was that patients were not attended to as they might be. If these stamps were properly taken up the difficulty would be removed. Last year they were the means of making £38,000, and double that amount might easily be obtained.

A DELEGATE: What is the amount required to maintain all the hospitals?

Mr. HOLLAND: That is rather a big question. The people of London contribute £6 per thousand, while those of Wigan give £58 per thousand and the people of Birmingham £30.

If we could bring London anywhere near these standards we should be all right.

A Delegate asked whether, if the scheme propounded by Mr. Holland became a great success, the public would have any voice in the management of the hospitals.

Mr. Holland said he considered it was absolutely right that those who subscribed to hospitals should manage them. In Poplar, continued Mr. Holland, the working men had almost entire control, but practically the hospital is entirely run by myself. (Laughter.)

Another Delegate remarked with regard to the stamps that the Committee of the Prince of Wales's Fund had decided to take no one into their confidence. They had resolved to distribute the money without consulting anyone outside their own body.

A Delegate eventually suggested that they had begun at the wrong end; what they wanted to know first was what the money was going to be subscribed for? The view commended itself to the meeting, and it was decided to adjourn the consideration of the subject of the better organisation of the sources of support to hospitals until the three other meetings arranged had taken place.

### CHARING CROSS HOSPITAL.

Lord Wantage, who presided at the annual court of governors of the Charing Cross Hospital, which was held at the hospital on Wednesday, the 16th inst., in moving the adoption of the annual report, said that there was a great deal in it which was matter of sincere congratulation. He first of all, however, had to deplore the loss of one of their treasurers, Mr. J. Biddulph Martin, who had been connected with the hospital since 1881, and for 13 years of that time had held office as treasurer. He next had to express the gratitude of the council for the liberal support the hospital had received from the public, as in spite of the numerous and heavy demands made on the public for charitable purposes, the year 1897 would be memorable in the history of the hospital as more successful financially than any of its predecessors. Speaking on the question of hospital abuse, he said that in order to ascertain how far the charge of abuse was justified in the case of Charing Cross Hospital, the council on two separate occasions, for four weeks at a time, deputed an efficient officer to make inquiries. The result was that of 1,071 patients who were interviewed only two were found to be unfit cases for hospital treatment, while there were 13 doubtful cases. The council were of opinion after the experiment that the charges of abuse made against hospitals were greatly exaggerated. He had visited the proposed site of the new out-patient department—the ground on which Toole's Theatre had stood—and he was greatly pleased to see what an excellent site and how eminently suited to the wants of the hospital it was. The money for this site had already been paid, and the hospital was now looking to the public to place the funds at their disposal to enable them to commence building operations.—Mr. J. S. Pearce seconded the report, which was unanimously adopted.—Votes of thanks to the various committees and officials, and the re-election of the retiring members of the council and the auditors completed the business of the meeting.

### CITY OF LONDON LYING-IN HOSPITAL.

The annual Court of Governors was held at the hospital on Wednesday, February 16th. Mr. W. Nicholson presided, and there were about a dozen governors present. It was reported that the past year had been satisfactory both as regards the financial and sanitary condition of the hospital. The total receipts amounted to £4,498 5s. 6d., and the expenditure to £4,415 5s. 6d., the latter including the purchase of India Three per Cents. to the extent of £551 12s. 3d.

As regards the work of the hospital, 521 women were delivered in the institution, being 28 more than in 1896, and the highest number since 1863. The number of children born was 524, namely 279 boys and 245 girls; three women had twins; 29 children were still-born; 15 were apparently still-born but restored; and 3 women and 15 children died. None of the maternal deaths was from septic causes. The first case was brought to the hospital by two medical men, who after some hours' attendance had failed to accomplish delivery, and the patient died from exhaustion a month after confinement. The second case was sent in a cab by a medical man; she was in a state of unconsciousness due to convulsions, from which she never recovered. The third was that of a woman who was attacked with severe hæmorrhage at her own home, and arrived at the hospital in a state of collapse from which she died a few hours after she was delivered.

The women delivered at their own homes numbered 1,668; 1,686 children were born, including 18 cases of twins; 69 were still-born, and 29 apparently so, but subsequently recovered. Six women and 32 children died. There were 44 midwives and 140 monthly nurses trained during the year, and of the 38 midwives who presented themselves for the London Obstetrical Society's examination 36 passed and received certificates.

An interesting discussion arose out of a question by Mr. Russell, a governor, as to whether the sphere of the institution's influence could not be extended. Mr. Owthwaite, the secretary, intimated that every case that came to the hospital was taken in, and as regards the out-patients only two-thirds of the tickets issued were used. This brought up one or two clergymen, who declared that their tickets were soon exhausted, and it was eventually decided to refer the matter to the committee to see if something could be done to ensure the tickets being more effectively made use of.

The reports having been adopted, an alteration was made in the bye-laws making the governors on the committee 16 instead of 12. Law 28 was also altered to read "The surgeon-accoucheur shall be a duly qualified practitioner holding separate diplomas in medicine and surgery, both of which diplomas shall be registered by the General Council of Medical Education prior to the day of election." The idea is to widen the field from which candidates may be chosen, the old law reading "The surgeon-accoucheur shall be a fellow or member of the Royal College of Surgeons of England, &c." Dr. Yarrow, the present holder of the office, was unanimously re-elected, and the Rev. G. H. Perry, the new rector of St. Luke's, Major Lewis Barned, Mr. W. K. Marriott, and Mr. J. C. Nicholson were added to the committee, while Messrs. Andrew P. Motion and John Paterson were elected trustees in the place of the Rev. W. G. Abbott, deceased, and Mr. T. Hillhouse, who has resigned.

### THE ROYAL SEA-BATHING INFIRMARY.

A special Court of Governors was held at the offices, Charing Cross, on Friday, February 18th, to consider various important proposals affecting the scope and administration of the institution. Hitherto, it should be explained, the court of directors has consisted of the president, the vice-presidents, treasurer, and twenty-four elected members, of whom nine had to be resident in the neighbourhood of Margate. These Margate members formed an executive committee, and were responsible for the management of the infirmary in accordance with the laws and the resolutions of the courts of governors and of directors.

Mr. Michael Biddulph, M.P., who presided over a small attendance, said that for a long time past things had not been going on satisfactorily, and while he did not attribute this to any individual shortcoming, it was apparent that the system of dual control was a bad one. In former days, when communication with Margate was slow and difficult, it was desirable to have the committee on the spot, but now that railway communication had made it possible to run down there in a very short time, it was felt that one governing body in London was more likely to conduct the business of the hospital satisfactorily than two authorities. Mr. Biddulph wished it to be clearly understood that he had not a single word to say against the committee at Margate, and he might add that the committee themselves realised that the system of dual control was a mistake, and had voted for its abolition in accordance with the views of the executive. It was proposed in addition to alter the title of the institution to that of "The Royal Sea-Bathing Hospital," since the word infirmary had become closely associated with poor-law relief; and to widen the law regulating the admission of patients, so that instead of being restricted to scrofulous diseases, the advantages of the hospital should be open to patients suffering from tubercular and other diseases, which, in addition to skilled medical and surgical attention, require the benefits of sea-air and sea-bathing for their successful treatment. The alteration would bring the institution more into accord with the intentions of the founder, the present form of law rigidly restricting the use of the hospital to cases of scrofula being of later introduction.

Mr. WEIGALL said he felt himself in a very difficult position, for while he did not wish to put any opposition in the way, he must enter his protest against the sweeping changes proposed. He must also take exception to the remark that the committee voted for their own abolition. They thought dual control a mistake, but they did not think they ought to be abolished in that way.

The CHAIRMAN, in reply, remarked that the committee voted for their abolition as a committee, but not as individuals. "We look confidently," he added, "to having members of our Margate committee on the committee of management."

The resolutions were then adopted, and their effect, in addition to what has been referred to above, will be to reduce the number of the elected members of the court of directors to 18, who will meet monthly. The court will be empowered to appoint a Finance Committee and also a Committee of Management, consisting of seven members and the treasurer. This latter committee, which will have absolute powers of management, will meet twice a month—once in London and once in Margate. In future, also, there will be one superintendent, who will be responsible to the committee. His duties will be administrative only, and the fact of a candidate having a medical qualification will not be a bar to his selection. In regard to the office of superintendent, Mr. BIDDULPH explained that it was considered desirable to appoint a man of higher social standing in future, and within the last few days they had seen a gentleman—Commander Gray, R.N.—who seemed a desirable candidate in every respect.

After the termination of the formal business, the CHAIRMAN announced that the hospital was about to be closed for repairs. The drains, it appears, are in need of attention, and before they can be opened it is necessary that the hospital should be cleared of patients. The expense involved, which will be considerable, has been more than covered by a donation from a gentleman connected with the charity. It should be said that no illness has occurred through the condition of the drains.

